# Cholinergic Stimulation Prevents the Development of Autoimmune Diabetes: Evidence for the Modulation of Th17 Effector Cells via an IFNγ-Dependent Mechanism

**DOI:** 10.3389/fimmu.2016.00419

**Published:** 2016-10-13

**Authors:** Junu A. George, Ghada Bashir, Mohammed M. Qureshi, Yassir A. Mohamed, Jamil Azzi, Basel K. al-Ramadi, Maria J. Fernández-Cabezudo

**Affiliations:** ^1^Department of Biochemistry, College of Medicine and Health Sciences, United Arab University, Al-Ain, UAE; ^2^Department of Medical Microbiology & Immunology, College of Medicine and Health Sciences, United Arab University, Al-Ain, UAE; ^3^Renal Division, Transplantation Research Center, Brigham and Women’s Hospital, Harvard Medical School, Boston, MA, USA

**Keywords:** neuroimmunology, inhibition of AChE, acetylcholine, IFNγ, Th17, type I diabetes

## Abstract

Type I diabetes (T1D) results from T cell-mediated damage of pancreatic β-cells and loss of insulin production. The cholinergic anti-inflammatory pathway represents a physiological link connecting the central nervous and immune systems *via* vagus nerve, and functions to control the release of proinflammatory cytokines. Using the multiple low-dose streptozotocin (MLD-STZ) model to induce experimental autoimmune diabetes, we investigated the potential of regulating the development of hyperglycemia through administration of paraoxon, a highly specific acetylcholinesterase inhibitor (AChEI). We demonstrate that pretreatment with paraoxon prevented hyperglycemia in STZ-treated C57BL/6 mice. This correlated with a reduction in T cell infiltration into pancreatic islets and preservation of the structure and functionality of β-cells. Gene expression analysis of pancreatic tissue revealed that increased peripheral cholinergic activity prevented STZ-mediated loss of insulin production, this being associated with a reduction in IL-1β, IL-6, and IL-17 proinflammatory cytokines. Intracellular cytokine analysis in splenic T cells demonstrated that inhibition of AChE led to a shift in STZ-induced immune response from a predominantly disease-causing IL-17-expressing Th17 cells to IFNγ-positive Th1 cells. Consistent with this conclusion, inhibition of AChE failed to prevent STZ-induced hyperglycemia in IFNγ-deficient mice. Our results provide mechanistic evidence for the prevention of murine T1D by inhibition of AChE and suggest a promising strategy for modulating disease severity.

## Introduction

Type 1 diabetes (T1D) is a T cell-mediated autoimmune disease in which insulin-producing β cells of the pancreatic islets of Langerhans are selectively destroyed. The onset of the hyperglycemia is preceded by a preclinical phase of insulitis, characterized by infiltration by T and B lymphocytes, myeloid cells, and NK cells into the pancreatic islets. Although the etiology of T1D remains incompletely understood, development of disease is influenced by genetic and environmental factors, including viral infections, food antigens, toxins, and stress. The autoimmune response is initiated in genetically pre-disposed individuals when physiological islet remodeling (1), viral infections, or inflammatory cytokines (2) induce the death of β cells. The release of β cell-specific antigens induces the activation of inflammatory T cells, leading to insulitis and, ultimately, β cell destruction. Several experimental models have been developed to study T1D, including spontaneous [biobreeding rats (BB); non-obese diabetic mice (NOD)] and chemically induced disease [multiple low-dose streptozotocin (MLD-STZ); alloxan] (3). In the MLD-STZ mouse model, many studies have shown that the destruction of β cells and development of hyperglycemia resembles the development of T1D in humans in being mediated by inflammatory T cells (both CD4 and CD8) and regulated by inflammatory cytokines (4–7). Moreover, this model has the advantage over the NOD model in that it induces only autoimmune diabetes and not any other systemic autoimmune disease.

It is well established that the nervous system plays a role in the regulation of immune responses and vice versa (8). The capacity of the cholinergic system to modulate immune responses has been amply demonstrated (9–11). Structurally, it has been shown that the major cholinergic parasympathetic nerve, the vagus nerve, innervates many organs, including GI tract, pancreas, and lymphoid tissues where nerve terminals form synaptic contacts with lymphoid cells. Muscarinic and nicotinic acetylcholine receptors (AChR) are known to be expressed on many cell types of the immune system, including lymphocytes, macrophages, and dendritic cells (12–14), suggesting that acetylcholine (ACh) may act as a neuroimmunomodulator in interactions between the nervous and immune systems. T lymphocytes express muscarinic (mAChR) and nicotinic acetylcholine receptors (nAChR) and synthesize ACh and acetylcholinesterase (AChE) (10). Functional studies have demonstrated that ACh stimulation of T cells through the α7 subunit of nAChR reduces mitogen-induced proliferation and secretion of proinflammatory cytokines (15). Likewise, activation of α7 nAChR on macrophages inhibits the secretion of TNF-α (9). In contrast, stimulation through mAChR induces the secretion of proinflammatory cytokines (16). These findings gave rise to the concept of the cholinergic anti-inflammatory pathway or inflammatory reflex (17), amply demonstrated to counteract endotoxemia-induced inflammation (18–20). In this reflex pathway, the presence of inflammatory molecules in the periphery stimulate the afferent vagus nerve that relay the signal to the brain which regulates, through the efferent vagus nerve, the production of proinflammatory cytokines (21). Our group previously demonstrated that inhibition of AChE promotes survival of mice following a lethal oral infection with *Salmonella*, highlighting the physiological significance of this pathway in mucosal immunity (11). Furthermore, cholinergic stimulation has been shown to decrease the severity of inflammatory conditions, such as experimental autoimmune encephalitis (EAE) (15), experimental autoimmune myasthenia gravis (22), and Alzheimer’s disease (23).

Several lines of evidence from human studies as well as animal models of spontaneous (NOD mice) and chemically induced (MLD-STZ) T1D indicate the crucial role played by Th17 cells in the pathogenesis of autoimmune diabetes (24–33). In addition to IL-17A, the signature cytokine produced by the Th17 lymphocyte subset, these cells also secrete a host of other inflammatory mediators including IL-17F, IL-21, IL-22, TNFα, GM-CSF, and IL-6 (34), which collectively drive the associated immunopathology. In contrast, induction of IFNγ in NOD mice protected against diabetes development by suppressing Th17 activity and inhibiting IL-17 production (24, 35). Therefore, IL-17 and IFNγ appear to play diverse and often cross-regulatory functions during the development of T1D. Given the central role of T cells and macrophages in the development of T1D and the existence of cholinergic innervation in the pancreas, we investigated the potential immunomodulatory effect of AChE inhibition on the development of diabetes using the MLD-STZ mouse model. Using paraoxon as a systemic AChE inhibitor (11), we demonstrate that cholinergic activation prevents the development of hyperglycemia by inhibiting pancreatic islet inflammation and β cell loss. Moreover, inhibition of AChE prevents the differentiation of naïve CD4^+^ T cells into IL-17-producing Th17 cells and, instead, promotes their differentiation to IFNγ-secreting Th1 cells. Interestingly, inhibition of AChE fails to modulate streptozotozin (STZ)-induced hyperglycemia in IFNγ-deficient (IFNγ^−/−^) mice, demonstrating the crucial role played by IFNγ in ACh-mediated inhibition of the autoimmune response against islet β cells. Our findings provide a rationale for a new strategy in the development of anti-diabetic therapies.

## Materials and Methods

### Experimental Animals

C57BL/6 mice were purchased from Harlan Olac (Bicester, United Kingdom) and bred in the animal facility of the College of Medicine and Health Sciences, UAE University. IFNγ^−/−^ mice were purchased from Jackson laboratories (USA) and generously provided by Dr Mariam Al-Shamsi at our institution. Female mice aged 8–10 weeks (weight range 20–22 g) were used for the experiments. All studies involving animals were carried out in accordance with, and after approval of, the animal research ethics committee of the College of Medicine and Health Sciences, UAE University.

### Chemicals

Paraoxon (Sigma, St. Louis, MO, USA), an organophosphorous compound, is a highly specific, irreversible, inhibitor of AChE (11). A stock solution (10 mmol/l) was prepared in acetone. Working solution for intraperitoneal (i.p.) injection was prepared *ex tempore* in pyrogen-free saline to a concentration of 80 nmol/ml. The final acetone concentration in the paraxon solution used for i.p. injection was ~108 μM. Each mouse received 40 nmol/day of paraoxon or saline in 0.5 ml volume. STZ (Sigma) was prepared *ex tempore* in citrate buffer (pH 4.5) and used i.p. at 60 mg/kg/day per mouse, unless otherwise indicated.

### Diabetes Induction

To induce autoimmune diabetes, the MLD-STZ model was used (3). C57BL/6 and IFNγ^−/−^ mice were administered five consecutive daily doses of STZ; control mice received citrate buffer. At different time points post-STZ administration, blood was drawn from the tail vein to determine glucose levels using *One-Touch-ultra-strip* (Lifescan, Zurich, Switzerland). Hyperglycemia was defined as non-fasting blood glucose >200 mg/dl.

### Experimental Protocol

Twenty age-matched mice were randomly assigned into two groups (10 animals per group). Group I served as control and received daily i.p. injection of sterile saline for 3 weeks. Group II mice received daily injection of paraoxon for 3 weeks. Mice were weighed weekly, at which time blood was also collected and analyzed for AChE activity. At the end of treatment, each group was divided randomly into two subgroups, A and B. Groups IA (Saline) and IIA (paraoxon) received daily injections of citrate buffer while groups IB (Saline/STZ) and IIB (paraoxon/STZ) received daily injection of STZ for five consecutive days. Pancreas, spleen, and serum were collected from mice sacrificed (ether exposure) at days 10 and 18, post-STZ administration. In some experiments, mice were followed for survival for up to 60 days.

### AChE Activity of Red Blood Cells

The detailed procedure for determining AChE enzyme activity in red blood cells (RBC) has been described (36). Briefly, freshly drawn, diluted, venous blood samples were incubated with DTNB (10 mM) and ethopropazine (6 mM) for 20 min at 37°C prior to addition of acetylthiocholine. The change in the absorbance of DTNB was measured at 436 nm. The AChE activity was calculated using an absorption coefficient of TNB^−^ at 436 nm (ε = 10.6 mM^−1^ cm^1^). The values were normalized to the hemoglobin (Hb) content (determined as cyanmethemoglobin) and expressed as mU/μM/Hb (37). All enzyme activities were expressed as percentage of the baseline activity (100%).

### Histology and Immunohistochemistry of Pancreatic Tissue

Excised pancreata were processed for histological analysis following established protocol (38, 39). Tissue sections were stained with hematoxylin and eosin (H&E), and images were captured using Olympus BX53 microscope equipped with digital camera DP26 (Tokyo, Japan). Indirect immunostaining for insulin was performed using guinea pig polyclonal antibody (Dako, Carpinteria, CA, USA) followed by FITC-conjugated donkey anti-guinea pig IgG (Jackson ImmunoResearch, West Grove, PA, USA). A three-step staining protocol was utilized to detect infiltrating T cells and macrophages. For T lymphocytes, CD3-specific rabbit polyclonal Ab (Dako) was used followed by biotinylated sheep anti-rabbit Ig (AbD Serotec, Hercules, CA, USA) and finally streptavidin-FITC (eBioscience, San Diego, CA, USA). For macrophages, we used rat F4/80 mAb (BMA Biomedicals, Switzerland) followed by streptavidin-HRP and DAB (Dako). Slides with fluorescence were counter-stained with propidium iodide (BD Biosciences, USA) and then examined and photographed under a Nikon C1 laser scanning confocal microscope. Slides stained with DAB were counter-stained with hematoxylin and visualized and photographed with an Olympus BX53. Histological quantification of CD3^+^ and F4/80^+^ cells was done on two to four non-consecutive sections per animal and using four to five mice per experimental group. All islets found in each section were included in the quantification, which was carried out in a blinded fashion.

### Antibodies and Flowcytometry

Analysis of spleen cells was carried out using a multi-color FACS analysis, following a standard procedure (39, 40). Cells were stained with a combination of directly conjugated mAbs, washed, and analyzed using FACSCanto II (BD Biosciences, San Jose, CA, USA). The antibodies used were CD3-FITC, CD4-APC, and CD8-APC-Cy7 (Biolegend, San Diego, CA, USA), CD19-PE-Cy7 and CD11c-PE (eBioscience), and CD11b-PE-Cy7 (BD Biosciences). Non-viable cells staining positive with 7AAD dye (eBioscience) were excluded from the analysis. Data collected on 50,000 cells were analyzed using FACSDiva software (BD Biosciences).

### Intracellular Cytokine Analysis

Spleen single cell suspensions were prepared and 2 × 10^6^ cells/ml were seeded in 24 well plates and stimulated with PMA (100 ng/ml)/Ionomycin (1 μg/ml) for 4 h at 37°C in the presence of brefeldin A (GolgiPlug; BD*cytofix/cytoperm plus^TM^* solution kit, BD Biosciences). After stimulation, cells were first stained with CD4-APC and CD8-APC-Cy7 antibodies (Biolegend), resuspended in fixation/permeabilization solution and finally stained with anti-IL-17A-PE and anti-IFNγ-PE-Cy7 antibodies (eBioscience) and run using FACSCanto II (BD Biosciences). Data collected from 50,000 cells were analyzed using FACSDiva software (BD Biosciences).

### Quantitative RT-PCR

qRT-PCR was carried out as previously described (40) on RNA extracted from spleen and pancreas from each animal. After RNA extraction and purification, cDNA was synthesized using Taqman reverse transcription reagents (Applied Biosystems, Foster City, CA, USA) following manufacturer’s protocol. TaqMan primers and probes were used to study the expression of the inflammatory markers IL-1β, IL-6, IL-12p40, IL-17A, IFNγ, and insulin (Applied Biosystems), and IL-23 and TNFα (Metabion, Germany) (Table 1). Transcript levels of target genes were normalized according to the dCq method to respective mRNA levels of the housekeeping gene HPRT.

**Table 1:** **List of primers**.

Gene	Taqman gene ID/sequence
IL-1β	Mm00434228-m1
IL-6	Mm00446190-m1
IL-12p40	Mm00434174-m1
IL-17F	Mm00521423-m1
IFN-γ	Mm01168134-m1
Insulin	Mm00731595-gh
TNFα (Metabion)	F: 5′-CCT CCC TCT CAT CAG TTC TAT-3′
	R: 5′-CTA GTT GGT TGT CTT TGA GAT CC-3′
	Probe: 5′-6-Fam-ACA AGC CTG TAG CCC ACG TCG TAG-BHQ-1-3′
IL-23p19 (Metabion)	F: 5′-CATGCTAGCCTGGAACG-3′
	R: 5′-GATCCTTTGCAAGCAGAA-3′
	Probe: 5′-6-Fam-TGACCCACAAGGACTCAAGGACA BHQ-1-3′

### Insulin and Cytokine Determination

Serum samples were assayed for insulin (Alpco diagnostics, Salem, NH, USA) level by sandwich ELISA and performed according to the manufacturer’s instructions. Assay sensitivity was 188 pg/ml.

### Statistical Analysis

Statistical significance between control and treated groups was analyzed using the unpaired, two-tailed Student’s *t*-test, using the statistical program of GraphPad Prism version 6 software. For multiple comparisons, we used One-way ANOVA with *post hoc* Tukey’s test (GraphPad Prism). Two-way ANOVA with Bonferroni post-test was used to analyze repeated measures data, as indicated. Survival analysis was performed by Kaplan–Meier survival curves and log-rank test, using the same GraphPad Prism program. Differences between experimental groups were considered significant when *p* values were <0.05.

## Results

### MLD-STZ-Induced Hyperglycemia

Studies were first carried out to optimize the STZ dose necessary to induce hyperglycemia (blood glucose >200 mg/dl) in C57BL/6 mice. Animals were divided into three groups, each receiving either 40, 50, or 60 mg/kg dose of STZ for five consecutive days. As can be seen, the percentage of mice developing hyperglycemia was 0, 50, and 100% by day 21 post administration of STZ at 40, 50, or 60 mg/kg, respectively (Image 1 in Supplementary Material). Hence, for all subsequent studies, a dose of 60 mg/kg STZ was used.

### Acetylcholinesterase Inhibition Prevents Hyperglycemia and Preserves Insulin Production

**Figure 1:**
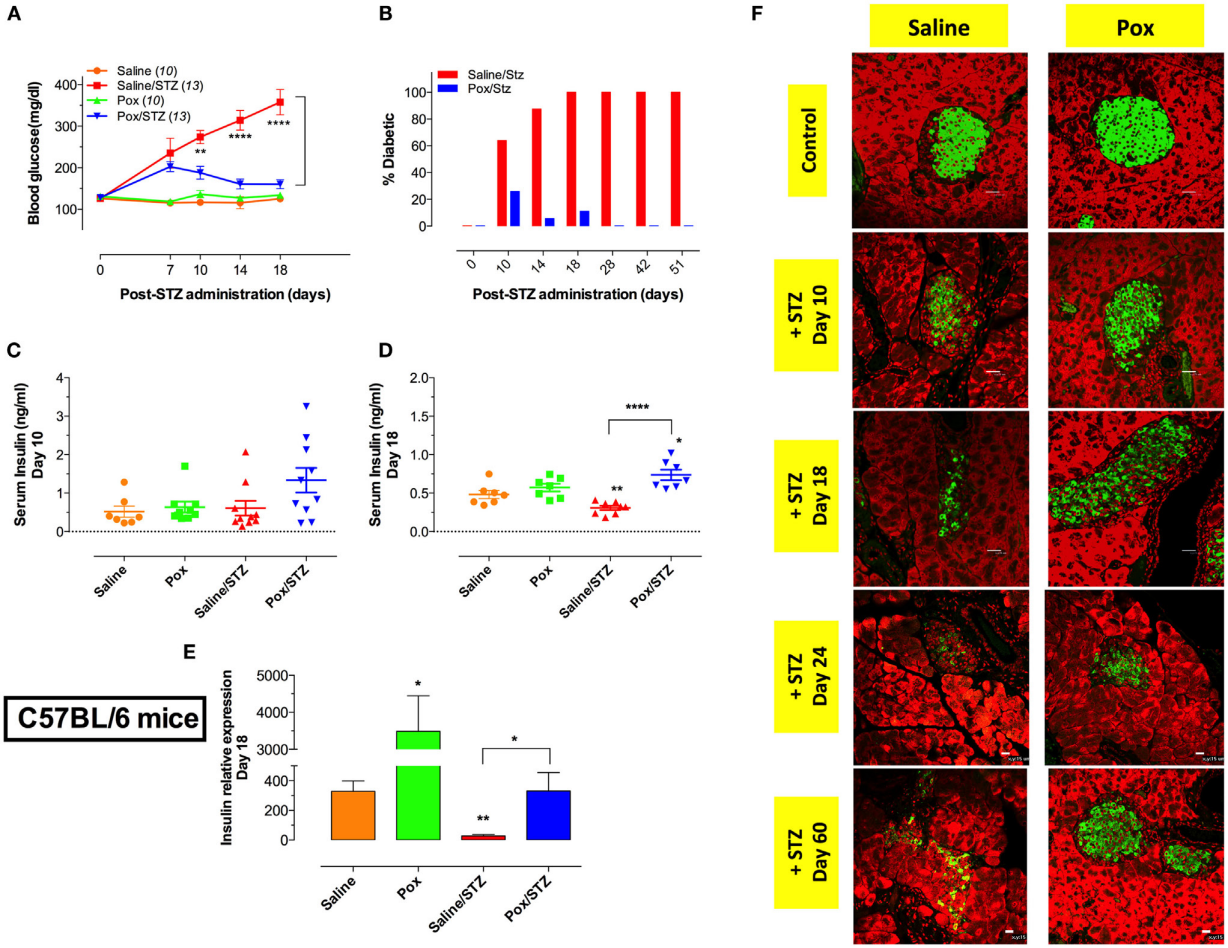
**Administration of AChE inhibitor modulates the development of STZ-induced diabetes**. C57BL/6 mice pretreated with paraoxon (Pox) or Saline for 3 weeks were challenged with 60 mg/kg/day of STZ for five consecutive days. **(A)** Blood glucose concentrations in tail blood samples were measured at indicated time points. Mice with blood glucose measurements >200 mg/dl were considered diabetic. Data are pooled from three independent experiments and the total number of mice is shown in parenthesis for each group. Two-way ANOVA with Bonferroni post-test was used for statistical analysis. **(B)** Percentage of diabetic animals in mice pretreated with saline or paraoxon followed by MLD-STZ challenge over an observation period of 51 days. Data are pooled from six independent experiments (22–25 mice per group up to day 18 post-STZ and 9 mice per group for later time points). **(C,D)** Serum insulin levels were determined at day 10 **(C)** and day 18 **(D)** post-STZ administration. **(E)** mRNA expression of pancreatic insulin in mice sacrificed at day 18 post-STZ treatment. Each data point represents the mean ± SEM of the values obtained from four to six animals per group. Student’s *t*-test was used for graphs **(C–E)**. Asterisks above bars denote statistically significant differences between the indicated experimental groups and saline-control group. Asterisks above brackets denote significance between the indicated experimental groups (*p < 0.05, **p ≤ 0.01, ***p ≤ 0.001, ****p ≤ 0.0001). **(F)** Light confocal micrographs (×40) of pancreatic islets of C57BL/6 mice pretreated with saline/STZ or Pox/STZ showing immunofluorescence of insulin-containing β cells. Total follow-up period was 60 days. Photos are representative of two individual experiments (four mice/group/experiment). Bar in the figures indicates 25 μm.

In order to investigate the potential effect of AChE inhibition on experimental diabetes, C57BL/6 mice were pretreated daily with paraoxon or saline for 3 weeks, and then given MLD-STZ (or saline), as described in the Section “Materials and Methods” and followed for the development of hyperglycemia. No changes in glucose levels were observed in control mice (saline or paraoxon only experimental groups) (Figure 1A). Saline-pretreated mice that received STZ developed progressive hyperglycemia, first evident at 7 days post-STZ administration, and that continued for up to 51 days (Figures 1A,B). All animals in this group were diabetic by day 18 post STZ administration (Figure 1B). In contrast, despite an initial mild elevation in blood glucose levels observed at day 7, mice pretreated with paraoxon prior to STZ were resistant to the development of hyperglycemia (Figure 1B). This remained evident for up to 51 days following STZ administration, the maximum period of observation (Figure 1B).

Serum insulin levels were determined on day 10 (Figure 1C) and 18 (Figure 1D) post STZ injection. Similar levels of serum insulin were observed in saline- and paraoxon-treated control mice at both time points (Figures 1C,D). Interestingly, for STZ-treated mice, while serum insulin was at normal level at day 10 post administration (Figure 1C), the level dropped significantly by day 18 to ~64% of control (Figure 1D). In contrast, no reduction in serum insulin was observed in mice pretreated with paraoxon followed by STZ (Figure 1D). The levels of serum insulin of paraoxon/STZ-treated mice tended to be higher than saline control at days 10 and 18 post STZ injection (2.6-fold and 1.6-fold increase, respectively; Figures 1C,D) with the difference being statistically significant at the latter date. Moreover, differences in serum insulin levels between saline/STZ and paraoxon/STZ groups at day 18 were highly significant and inversely correlated with serum glucose levels (compare Figures 1A,D).

Insulin mRNA expression was also analyzed in pancreatic tissue at day 18 post-STZ treatment (Figure 1E). A dramatic 10.6-fold increase in insulin expression was observed in paraoxon-treated mice compared with saline control (Figure 1E). In sharp contrast, STZ treatment (saline/STZ group) led to a 12-fold reduction in the level of insulin mRNA relative to saline control. In mice pretreated with paraoxon prior to STZ (paraoxon/STZ group), insulin mRNA levels were essentially similar to those observed in saline control group (Figure 1E). Immunohistochemical analysis of insulin-producing β cells in pancreatic tissue revealed a gradual loss of insulin positivity in STZ-treated mice (saline/STZ group), which was evident starting at day 10 following STZ administration and continued up to day 60 (Figure 1F). In contrast, mice pretreated with paraoxon (paraoxon/STZ group) showed evidence of islet preservation and protection from STZ-induced loss of β cells, albeit not total, up to day 60 (Figure 1F). These results demonstrate that inhibition of AChE increases the expression of insulin mRNA in pancreatic cells and prevents STZ-induced hyperglycemia.

### Inhibition of AChE Prevents Insulitis and Destruction of Islets Induced by MLD-STZ

**Figure 2:**
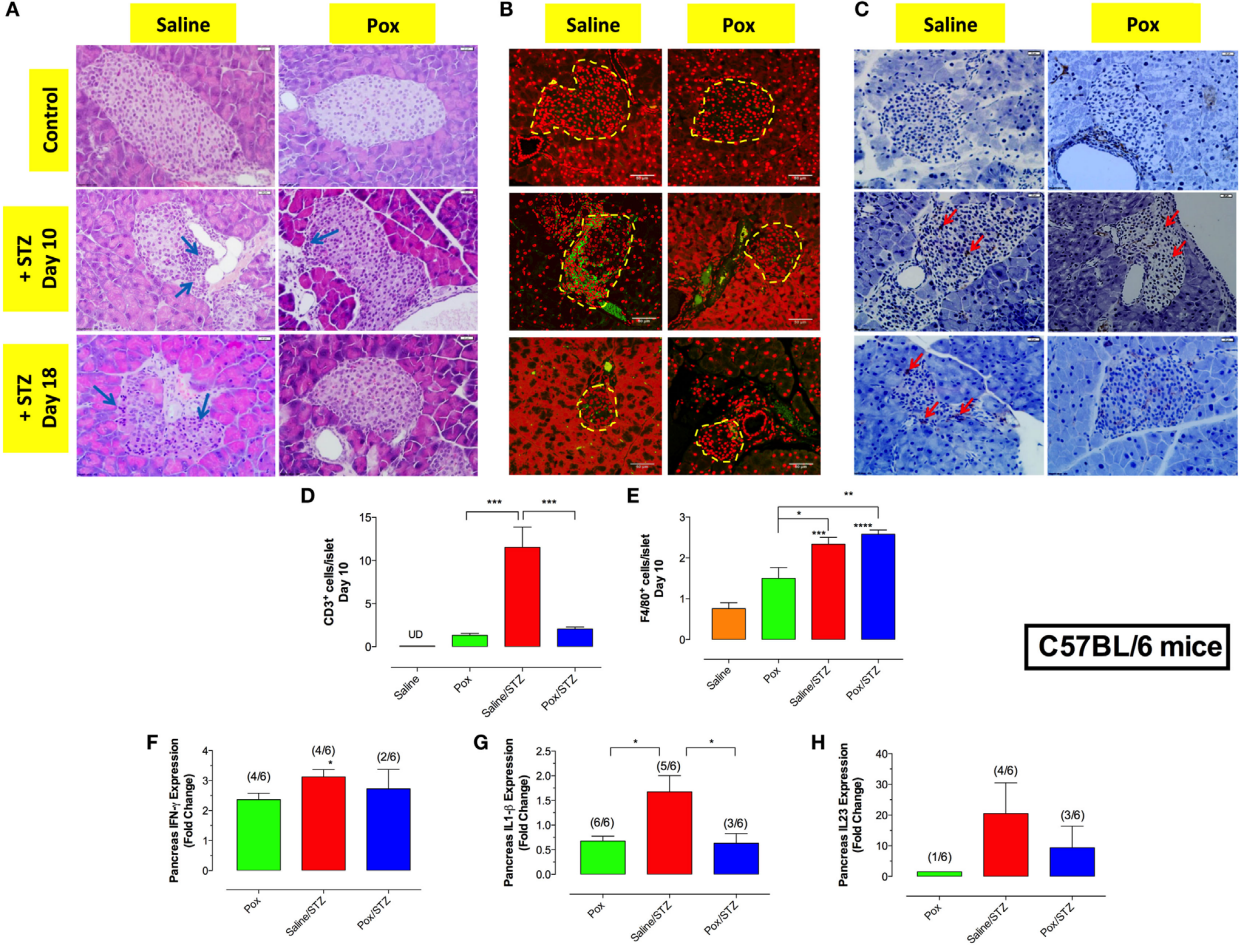
**Inhibition of AChE reduces STZ-mediated insulitis**. **(A)** Representative images of H&E stained pancreatic islets from the four different experimental groups sacrificed at day 10 or 18 post-STZ administration. Arrows indicate infiltrating inflammatory cells (bar = 20 μm). **(B,C)** Pancreatic sections were stained with anti-CD3 **(B)** or F4/80 **(C)** antibodies, as described in Section “Materials and Methods” to detect T cells and macrophages, respectively. Dashed lines delineate pancreatic islets. Representative images (40×) from two independent experiments (four mice/group/experiment) are shown. Arrows indicate representative cells. Bars in the figures indicate 50 μm in CD3 staining and 20 μm in F4/80 staining. **(D,E)** Quantitative estimation of the number of T cells **(D)** and macrophages **(E)** per islet in pancreatic section of different treatment groups sacrificed at day 10 post-STZ administration. Data are shown as the mean ± SEM of the number of positive cells per high power field. **(F–H)** Relative expression levels of IFN-γ **(F)** and IL-1β **(G)** and IL-23p19 **(H)** mRNA, expressed as fold change compared to saline controls, isolated from pancreas of mice sacrificed day 18 post-STZ administration. Each data point represents the mean ± SEM of the values obtained in that group. Since cytokine expression was not detectable in every mouse of each group, the ratio of the animals positive for each cytokine is also shown. Fractions above the bars indicate the number of mice with positive value/total number of mice in the group. One-way ANOVA was used for statistical analysis in Figures A–E. Asterisks above bars denote statistically significant differences between the indicated experimental groups and saline-control group. Asterisks above brackets denote significance between the indicated experimental groups (*p < 0.05; **p ≤ 0.01; ***p ≤ 0.001).

Hematoxylin and eosin staining of pancreatic tissue showed highly infiltrated islets in saline/STZ group at days 10 and 18 post-STZ administration (Figure 2A). Pretreatment with paraoxon reduced islet cell infiltration at day 10 (paraoxon/STZ group) and, by day 18, the pancreatic islets appeared completely healthy with no evidence of insulitis (Figure 2A). No infiltrating cells were observed in islets of saline or paraoxon control groups (Figures 2A–E). Immunohistological analysis of the islets at days 10 and 18 revealed the presence of CD3^+^ T cells (Figure 2B) and F4/80^+^ myeloid cells (Figure 2C) in STZ-treated mice. However, the degree of T cell infiltration was significantly reduced in mice pretreated with paraoxon (Figures 2B,D). At day 10, T cells were distributed uniformly throughout the islets of both saline/STZ and paraoxon/STZ groups. At day 18, however, only a few T cells located primarily in the periphery of the islets could be observed in paraoxon/STZ mice, while in saline/STZ group these cells were uniformly distributed within the islets (Figure 2B). Administration of STZ also induced a significant increase in intra-islet recruitment of macrophages, regardless of the pretreatment received (Figures 2C,E). No significant differences were found between saline and paraoxon control groups (Figure 2E).

Next, we investigated mRNA expression of proinflammatory cytokines in pancreatic tissue at day 18 post-STZ (Figures 2F,G). STZ administration led to a significant increase (3.1-fold) in IFNγ mRNA expression compared with saline controls (Figure 2F). Interestingly, mice pretreated with paraoxon alone also exhibited a 2.4-fold increase in pancreatic IFNγ expression compared with controls. In contrast, mice pretreated with paraoxon followed by STZ showed similar levels of IFNγ expression to those receiving paraoxon alone (detectable in only 2/6 mice examined; Figure 2F). Expression of pancreatic IL-1β was also assessed in the different experimental groups. The highest level of IL-1β expression was observed in saline/STZ group (Figure 2G). In contrast, IL-1β expression levels dropped significantly in paraoxon (2.5-fold) and paraoxon/STZ (2.7-fold) experimental groups compared with saline/STZ group (Figure 2G). For pancreatic IL-23, expression was almost undetectable in saline and paraoxon groups, was maximal in saline/STZ group and decreased by >50% in paraoxon/STZ-treated mice compared with saline/STZ group; however, this difference was not statistically significant (Figure 2H). Analysis of pancreatic TNFα expression revealed only background levels with no significant differences among the experimental groups (data not shown). Moreover, no cytokines could be detected in pancreatic tissue at day 10 post-STZ treatment in any of the groups (data not shown).

### Inhibition of AChE Reduced Splenic Proinflammatory Cytokines and Th17 Effector Cells

**Figure 3:**
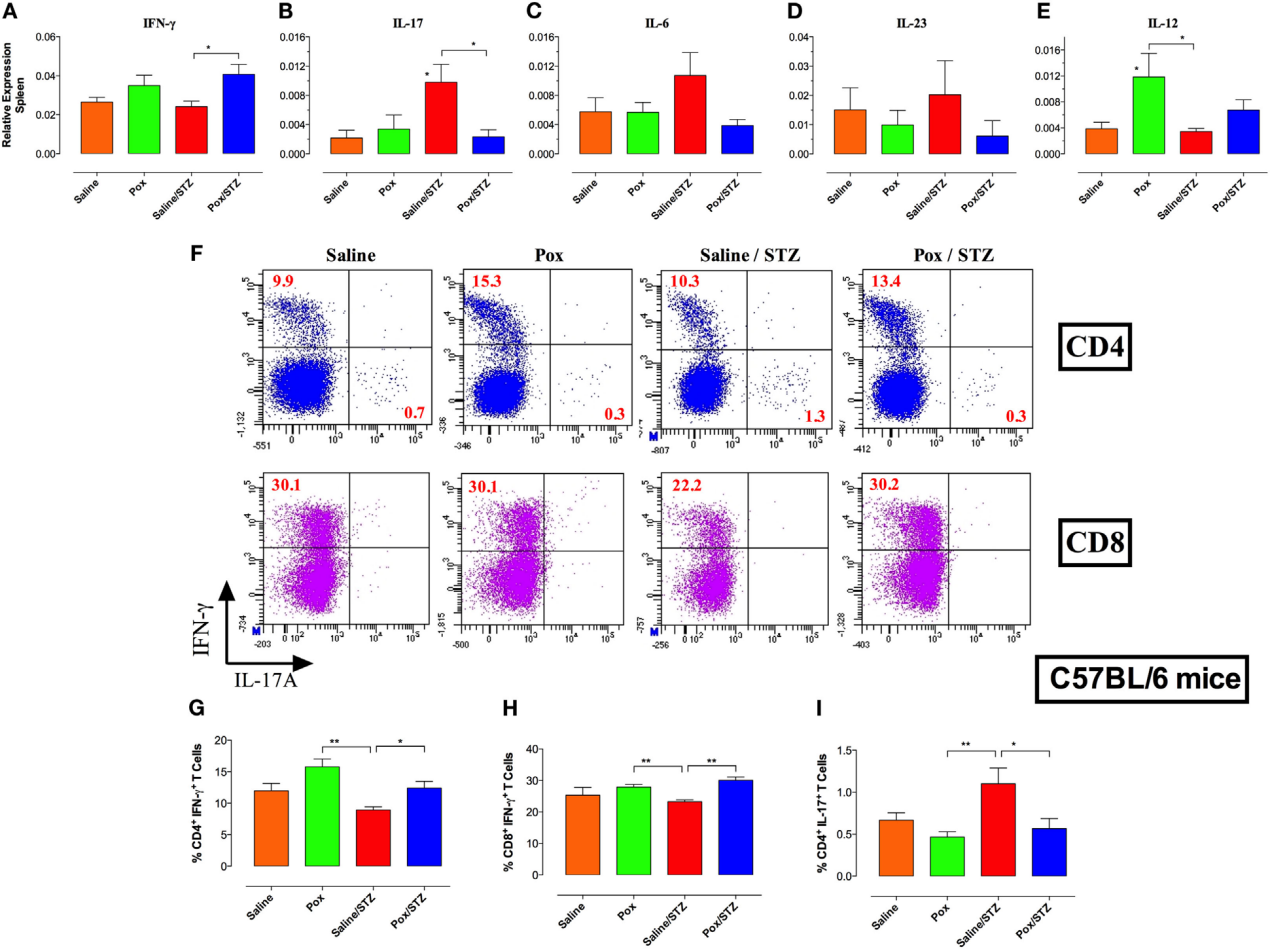
**Inhibition of AChE suppresses STZ-induced Th17 cells and promotes IFNγ-secreting Th1 differentiation**. C57BL/6 mice pretreated with paraoxon (Pox)/Saline for 3 weeks were administered STZ/vehicle for five consecutive days and sacrificed 18 days later. **(A–E)** Relative mRNA expression levels of IFNγ **(A)**, IL-17F **(B)**, IL-6 **(C)**, IL-23p19 **(D)**, and IL-12p40 **(E)** isolated from whole spleen. Each value represents the mean ± SEM of data collected from three independent experiments (7–10 mice/group). One-way ANOVA was used for the statistical analysis for **(A–E)** graphs. Asterisks above bars denote statistically significant differences between the indicated experimental groups and saline-control group. Asterisks above brackets denote significance between the indicated experimental groups (*p < 0.05). **(F–I)** Intracellular cytokine analysis. Splenocytes were stimulated *ex vivo* with PMA/ionomycin and analyzed for CD4 and CD8 positivity and IFNγ and IL-17 content. Each experimental group included three mice. **(F)** Representative results of individual mice are shown, and are representative of two independent experiments. Percentages of CD4^+^IFNγ^+^ **(G)**, CD8^+^IFNγ^+^ **(H)**, and CD4^+^IL-17A^+^ **(I)** T cells are depicted in the graphs. Values represent the mean ± SEM of six mice per group. Student’s *t*-test was used for data analysis of Figures G–I. Asterisks denote statistically significant differences between the indicated groups (*p < 0.05, **p ≤ 0.01).

Next, we explored the effect of AChE inhibition on the peripheral immune response in STZ-treated mice. Immunophenotyping revealed no gross differences in the percentage of major spleen cell populations among the four different experimental groups at either day 10 or 18 post STZ administration (Images 2 and 3 in Supplementary Material), the only exception being a small but significant decrease in the frequency of CD8^+^ T cells in the paraoxon/STZ group compared with saline/STZ-treated mice at day 10 post-STZ administration (Images 2 and 3 in Supplementary Material). Given the role of IL-17 and Th17 cells in autoimmune diabetes and their counter regulation by IFNγ (27, 41), mRNA expression levels of IFNγ and IL-17 were determined in spleen cells. Moderate upregulation of IFNγ expression was observed in mice pretreated with AChE inhibitor (paraoxon and paraoxon/STZ groups) compared with saline control (Figure 3A), with the differences being statistically significant between saline/STZ and paraoxon/STZ groups. Conversely, while administration of STZ led to a 4.7-fold increase in IL-17 expression compared with saline control animals (Figure 3B), pretreatment with paraoxon abrogated the IL-17 response (paraoxon/STZ group; Figure 3B). These results suggest that inhibition of AChE led to an increase in IFN-γ production and a reduction in STZ-induced IL-17. Further analysis revealed a reduction in the expression of IL-6 and IL-23, cytokines involved in the differentiation of Th17 cells, in groups receiving paraoxon compared with saline group (Figures 3C,D). Moreover, compared with control animals, IL-6 expression was upregulated in saline/STZ group (Figure 3C). On the other hand, expression levels of IL-12, an IFNγ-promoting cytokine, were found to be upregulated in paraoxon-treated groups compared with saline control (2.7-fold and 1.8-fold increase in paraoxon and paraoxon/STZ groups, respectively; Figure 3E). Significant differences in IL-12 expression were observed between paraoxon-treated mice compared with the saline or saline/STZ group (Figure 3E). No significant differences were observed in the expression of TNFα and IL-1β proinflammatory cytokines among experimental groups (data not shown). These findings suggest that inhibition of AChE may prevent STZ-induced hyperglycemia by modulating the balance between Th1 and Th17 development.

To assess this directly, we determined the percentage of IL-17^+^ and IFNγ^+^ splenic T cells at day 18 post-STZ administration. *Ex vivo* stimulation with PMA/ionomycin followed by intracellular cytokine staining revealed that pretreatment with paraoxon induced a moderate increase in the percentage of IFNγ^+^CD4^+^ cells (Figure 3F). Saline/STZ group exhibited significant reduction in percentage of IFNγ^+^CD4^+^ and IFNγ^+^CD8^+^, but increased percentage of IL-17^+^CD4^+^, T cells compared with paraoxon and paraoxon/STZ group (Figures 3F–I). Percentage of CD4^+^IL-17^+^ cells in saline/STZ group was significantly increased compared with paraoxon-treated groups (2.4-fold and 2-fold that of paraoxon and paraoxon/STZ groups, respectively) (Figures 3F,I). Expression of IL-17 was detected mainly in CD4^+^, but not CD8^+^ T cells (Figure 3F). These results support the idea that inhibition of AChE leads to a preferential induction of a Th1 response that suppresses STZ-driven Th17 cell differentiation.

### Inhibition of AChE Fails to Prevent STZ-Induced Hyperglycemia in IFNγ^−/−^ Mice

**Figure 4:**
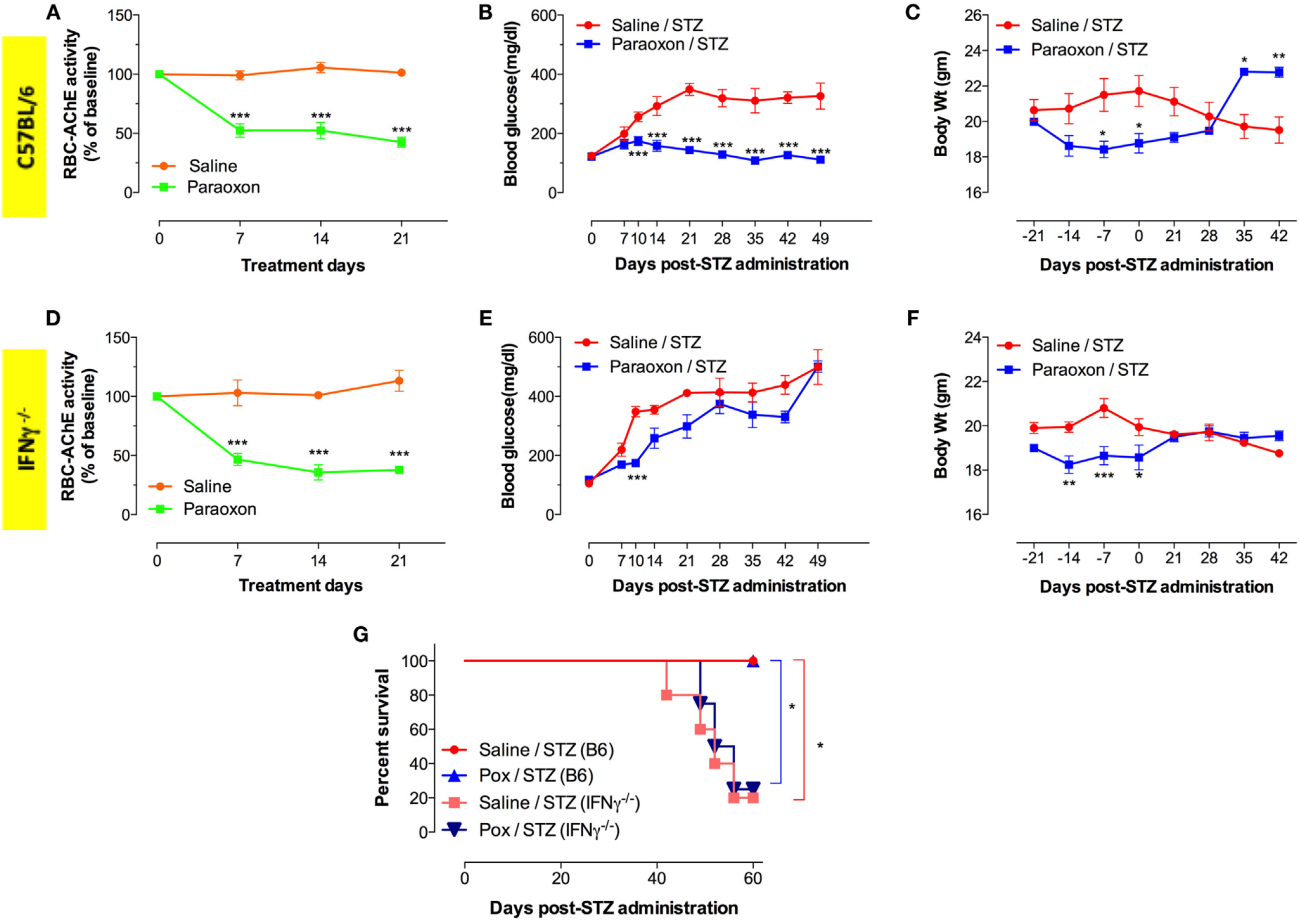
**IFNγ is required for amelioration of STZ-induced hyperglycemia**. Transient reduction in RBC-AChE activity in C57BL/6 **(A)** and IFNγ^−/−^ **(D)** mice during the course of paraoxon administration. All enzyme activities are expressed as percentage of the baseline activity (100%). Blood glucose levels in C57BL/6 **(B)** and IFNγ^−/−^ **(E)** mice were measured at indicated time points post STZ treatment. Changes in body weights in C57BL/6 **(C)** and IFNγ^−/−^ **(F)** mice during paraoxon treatment and up to 42 days post-STZ administration. The mean values ± SEM are depicted in all graphs. Two-way ANOVA with Bonferroni post-test was used for the statistical analysis of **(A–F)**. Asterisks denote statistically significant differences between the two groups at each time point (*p < 0.05, **p ≤ 0.01, ***p ≤ 0.001, ****p ≤ 0.0001). **(G)** Survival was followed for up to 60 days after STZ administration. Kaplan–Meier and log-rank test was used for analysis. All results represent cumulative data of two independent experiments. Asterisks denote statistically significant differences between the indicated groups (*p < 0.05).

To further elucidate the role of IFNγ in the development of hyperglycemia, we utilized the same MLD-STZ model to induce diabetes in IFNγ^−/−^ mice. Administration of paraoxon induced 50–60% inhibition in AChE activity in both wild-type (WT) C57BL/6 as well as IFNγ^−/−^ mice (Figures 4A,D). Importantly, pretreatment with paraoxon protected against STZ-triggered hyperglycemia in WT (Figure 4B) but not in IFNγ^−/−^ mice (Figure 4E). WT mice developed hyperglycemia 7–10 days post STZ administration and this continued up to 7 weeks later (Figure 4B). In contrast, no hyperglycemia was observed in paraoxon-pretreated WT mice after STZ injection (Figure 4B). It is intriguing to point out that paraoxon pretreatment in IFNγ^−/−^ mice did appear to protect against onset of hyperglycemia during the first 10 days following STZ administration (Figure 4E). Subsequently, however, paraoxon-pretreated IFNγ^−/−^ mice became hyperglycemic and, by day 49, their blood glucose levels reached 500 mg/dl, identical to saline-pretreated mice (Figure 4E). A moderate degree of weight loss (10–15%) is commonly observed in mice treated with AChE inhibitors (36, 42), and this was observed in both WT and IFNγ^−/−^ mice particularly during the 3-week (day –21 to day 0) paraoxon pretreatment period (Figures 4C,F). Following the end of paraoxon pretreatment period, the animals’ weights gradually normalized and by day 28 were identical to saline-pretreated mice (Figures 4C,F). As WT mice developed hyperglycemia due to STZ, they begin to loose weight and this was clearly evident by day 35 post STZ injection (Figure 4C). However, paraoxon-pretreated WT mice exhibited no further loss of weight after STZ and, in fact, their weights were higher than saline/STZ group at days 35 and 42 post STZ (Figure 4C). In contrast, although IFNγ^−/−^ mice also recovered their initial weight after paraoxon treatment, they did not exhibit further weight gain after STZ administration due to their hyperglycemic state (Figures 4E,F). Finally, we also assessed long term survival in STZ-injected mice (C57BL/6 and IFNγ^−/−^) following pretreatment with either saline or paraoxon (Figure 4G). Irrespective of the treatment, no deaths were recorded in C57BL/6 mice up to 60 days post STZ administration. In sharp contrast, only about 20% of IFNγ^−/−^ mice, whether pretreated with saline or paraoxon, survived by day 60 post-STZ (Figure 4G). This is most likely due to the higher levels of hyperglycemia observed in these mice (Figure 4E). These findings confirm the important role that IFNγ plays in cholinergic pathway-mediated inhibition of diabetes development in the MLD-STZ model.

### AChE Inhibition Does Not Prevent STZ-Mediated Islet β Cell Damage in IFNγ^−/−^ Mice

**Figure 5:**
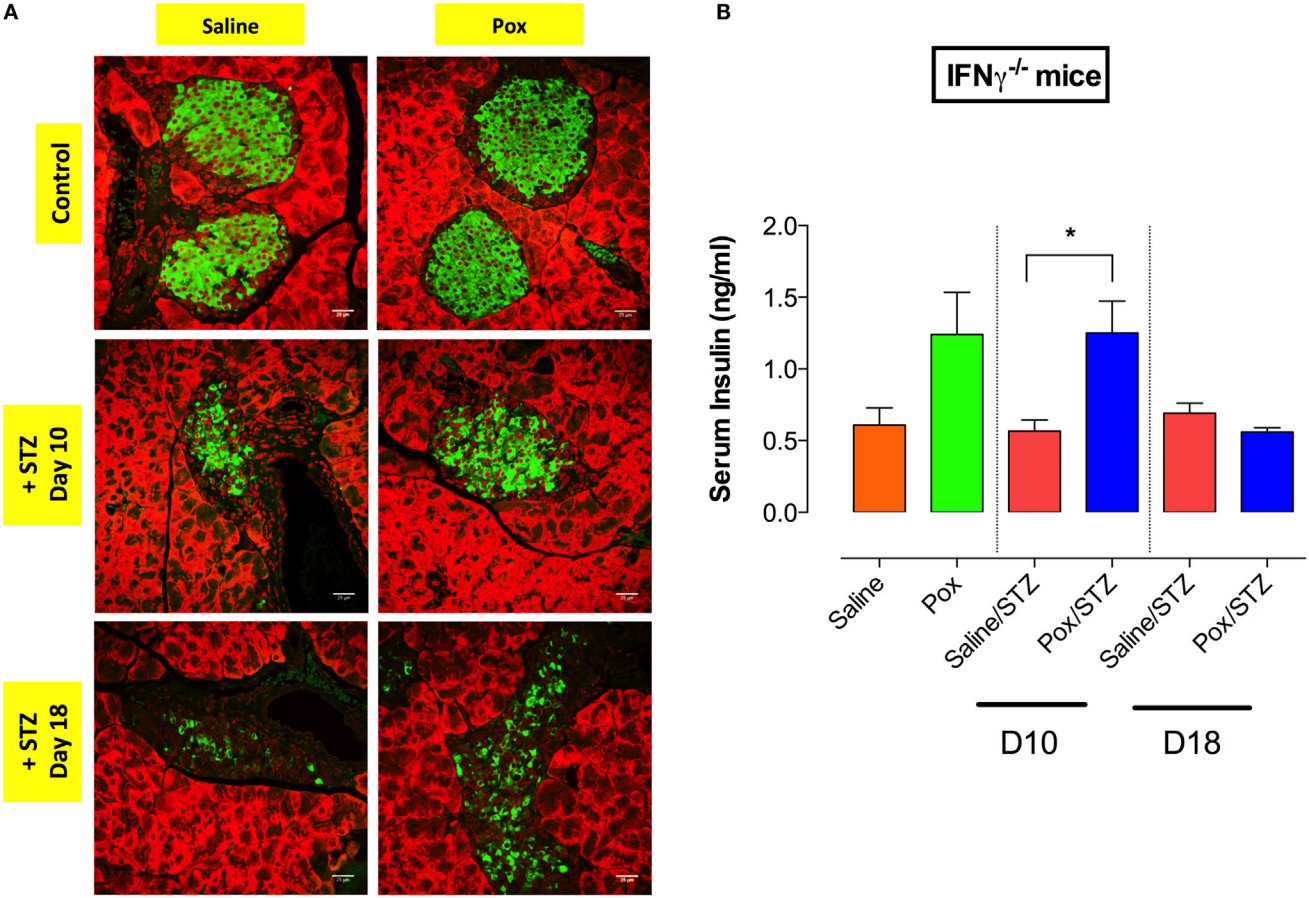
**Inhibition of AChE fails to prevent the loss of insulin-producing β cells induced by STZ administration in IFNγ^−/−^ mice**. IFNγ^−/−^ mice treated with paraoxon (Pox) or Saline for 3 weeks were challenged with 60 mg/kg/day of STZ for five consecutive days. **(A)** Light confocal micrographs (40×) of pancreatic islets from IFNγ^−/−^ mice from different experimental groups at days 10 and 18 post-STZ, showing immunofluorescence to insulin-containing β cells. Photos are representative of two individual experiments (four mice/group/experiment). Bar in the figures indicates 25 μm. **(B)** Serum insulin levels were determined at day 10 and 18 post-STZ administration. Student’s *t*-test was used for the statistical analysis. Asterisks denote statistically significant differences between experimental groups (*p < 0.05).

Next, we analyzed the status of insulin-producing cells in pancreatic tissue of IFNγ^−/−^ mice at days 10 and 18 post STZ administration. Compared with saline controls, saline/STZ group showed a reduction in insulin-producing β cells by day 10 post STZ injection, and this became more pronounced at day 18 post STZ (Figure 5A). Pretreatment with paraoxon appeared to delay the loss of insulin-positive β cells. Moreover, paraoxon-treated IFNγ^−/−^ mice exhibited higher levels of serum insulin compared with saline control (Figure 5B; paraoxon group). No reduction in serum insulin was observed in paraoxon/STZ-treated mice during the first 10 days post-STZ administration (paraoxon/STZ group). However, by day 18, serum insulin levels dropped to become similar to the ones in the saline/STZ group (Figure 5B). These data suggest that IFNγ does not play a role in the initial control of STZ-induced hyperglycemia.

**Figure 6:**
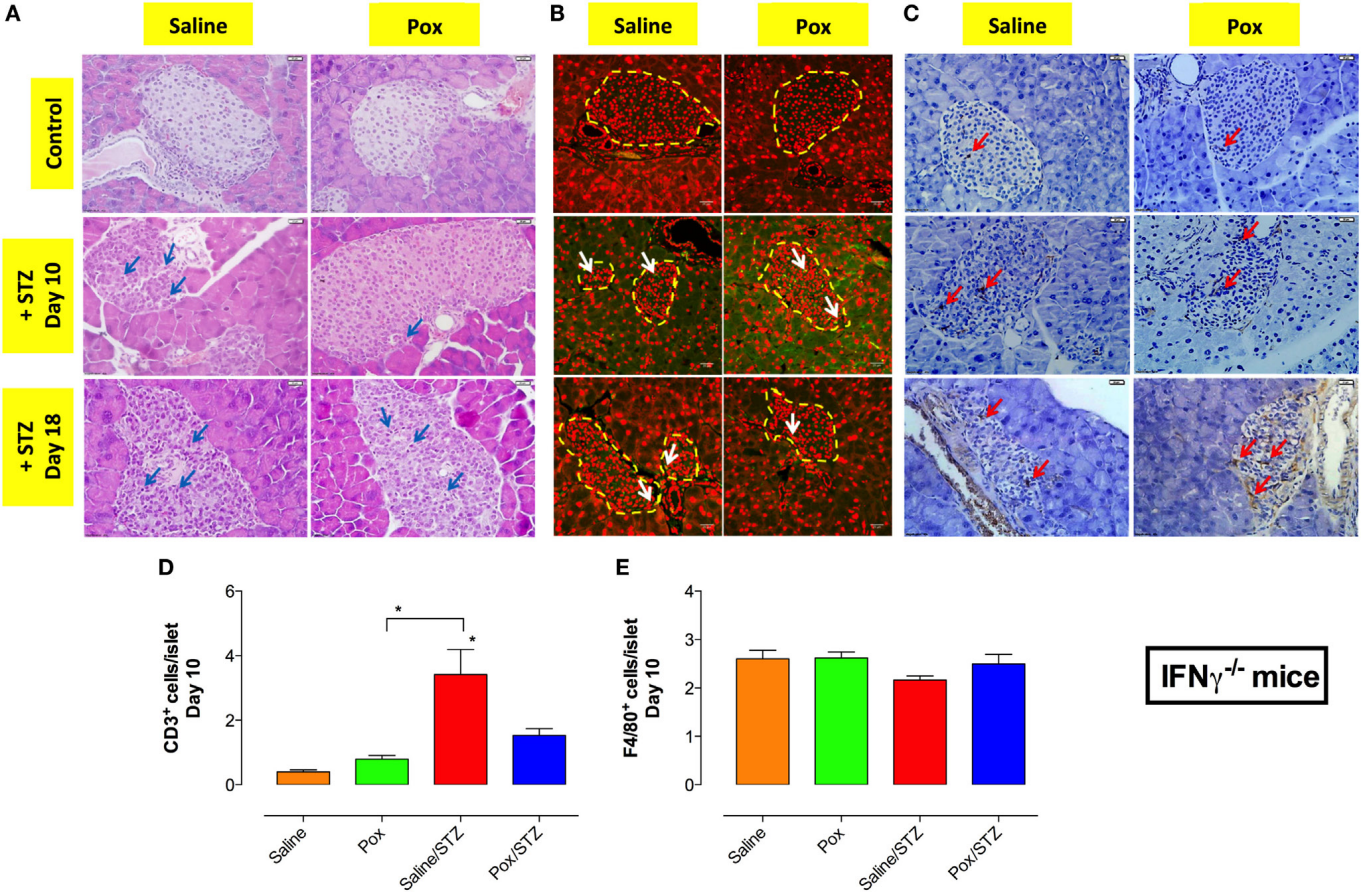
**In IFNγ^−/−^ mice, inhibition of AChE does not prevent STZ-induced insulitis**. **(A)** Paraffin embedded pancreata from the four different experimental groups, sacrificed at day 10 or 18 post-STZ administration, were sectioned and stained with H&E. Representative images are shown where arrows indicate infiltrating inflammatory cells (bar = 20 μm). Pancreatic sections were also stained with anti-CD3 **(B)** or F4/80 **(C)** antibodies, as described in Section “Materials and Methods” to detect T cells and macrophages, respectively. Dashed lines delineate pancreatic islets. Representative images (40×) from two independent experiments (four mice/group/experiment) are shown. Arrows indicate representative cells. Bars in the figures indicate 50 μm in CD3 staining and 20 μm in F4/80 staining. Quantitative estimation of the number of T cells **(D)** and macrophages **(E)** per islet in pancreatic section of different treatment groups sacrificed at day 10 post-STZ administration. Data are shown as the mean ± SEM of the number of positive cells per high power field. One-way ANOVA was used for the statistical analysis **(D,E)**. Asterisks above bars denote statistically significant differences between the indicated experimental groups and saline-control group. Asterisks above brackets denote significance between the indicated experimental groups (*p < 0.05).

Hematoxylin and eosin staining of pancreatic tissue of IFNγ^−/−^ mice showed highly infiltrated islets in the saline/STZ group at day 10 and 18 post STZ administration (Figure 6A; left middle and bottom panels). In contrast, pancreatic islets from paraoxon/STZ group exhibited mild infiltration at day 10, but by day 18 all islets appeared infiltrated although to a lesser extent than in saline/STZ group (Figure 6A; right middle and bottom panels). No apparent cell infiltration could be observed in islets of saline or paraoxon control groups (Figure 6A; top panels). Immunohistological analysis of the islets at day 10 and 18 revealed the presence of fewer CD3^+^ T cells than in WT mice, with highest levels being detected in saline/STZ group (Figures 6B,D). Pretreatment with paraoxon reduced by >60% the extent of T cell infiltration (paraoxon/STZ group) compared to saline/STZ group (Figure 6D). Staining with F4/80 mAb revealed a similar extent of macrophage infiltration in all four experimental groups (Figures 6C,E).

### Inhibition of AChE Leads to a Partial Alteration in Th17 Cell Ratio in the Absence of IFNγ

**Figure 7:**
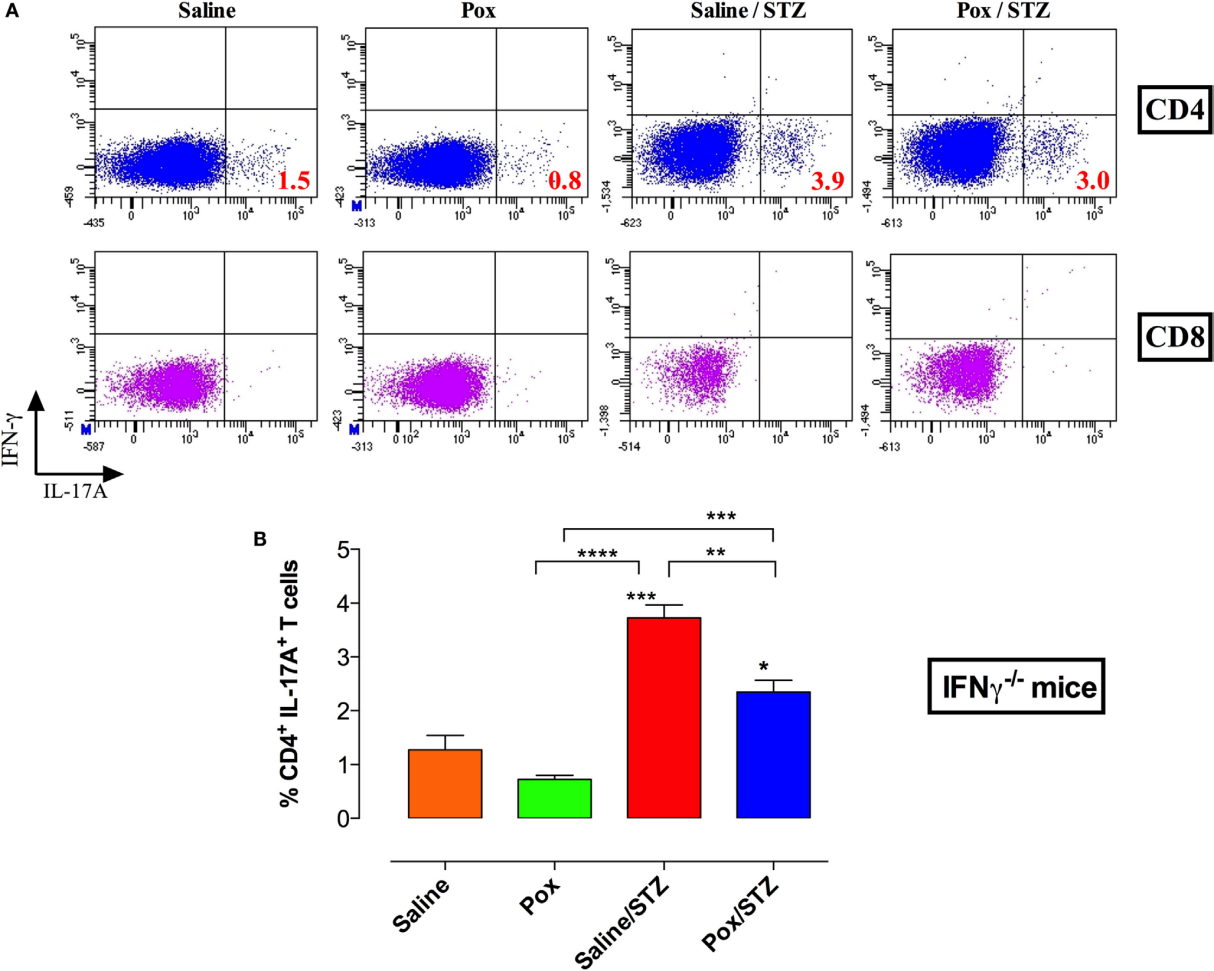
**Inhibition of AChE in the absence of IFNγ production leads to a partial decrease in Th17 differentiation**. IFNγ^−/−^ mice treated with paraoxon (Pox) or Saline for 3 weeks were administered STZ or vehicle for five consecutive days and sacrificed 18 days later. Spleens were obtained and single cell suspensions prepared. Splenocytes were stimulated with PMA/ionomycin and analyzed for CD4/CD8 positivity and IFNγ/IL-17 content **(A)**. Each experimental group included three mice. Results of individual mice are shown and are representative of two independent experiments. **(B)** Percentages of CD4^+^IL-17A^+^ T cells are depicted. Values represent the mean ± SEM of three mice per group. Data analysis was done by Student’s *t*-test. Asterisks above bars denote statistically significant differences between the indicated experimental groups and saline-control group. Asterisks above brackets denote significance between the indicated experimental groups (*p < 0.05, **p ≤ 0.01, ***p ≤ 0.001, ****p ≤ 0.0001).

As expected, intracellular staining of splenocytes of IFNγ^−/−^ mice showed no CD4^+^IFNγ^+^ or CD8^+^IFNγ^+^ T cells (Figure 7A). AChE inhibition induced a partial but not statistically significant reduction in the percentage of CD4^+^IL-17^+^ cells (paraoxon group). STZ administration induced the differentiation of CD4^+^IL-17^+^ cells in saline/STZ group (mean ± SEM = 3.7 ± 0.2%; Figures 7A,B) which represents a 2.9-fold increase in comparison to saline controls. For paraoxon/STZ-treated mice, the percentage of CD4^+^IL-17^+^ cells was slightly, but significantly, reduced (2.3 ± 0.2%) compared to saline/STZ group but was still significantly higher than that of saline and acetylcholinesterase inhibitor (AChEI) groups (Figures 7A,B). Nevertheless, the presence of a significant level of CD4^+^IL-17^+^ T cells in paraoxon/STZ-treated IFNγ^−/−^ mice is in sharp contrast to the findings in WT mice where similar treatment reduced the ratio of CD4^+^IL-17^+^ T cells to background levels (Figure 3F). These results highlight the immunomodulatory effect of the cholinergic pathway and the important role of IFNγ in preventing the development of STZ-induced diabetes.

## Discussion

Several studies have revealed the importance of the parasympathetic nervous system in regulating immune responses in infectious (9, 11) as well as autoimmune disease models (15, 22). The purpose of this study was to explore the effect of AChE inhibition on the onset and development of T1D induced by MLD-STZ administration. Cholinergic stimulation was induced through the utilization of an irreversible specific inhibitor of AChE, the primary ACh-hydrolyzing enzyme, thus leading to increased ACh levels. The findings of the current study demonstrate four main points. First, prophylactic cholinergic stimulation inhibits development of STZ-mediated hyperglycemia and T1D. Second, this is achieved by direct enhancement of insulin production by pancreatic β cells and by protecting these cells against STZ-induced autoimmune, cytotoxic damage. Third, ACh induces a shift in MLD/STZ-triggered T cell differentiation away from pathogenic IL-17-secreting Th17 cells to IFN-γ-secreting Th1 cells. Finally, ACh-mediated protection is significantly attenuated in mice deficient in IFNγ synthesis, providing strong evidence for the critical role of this cytokine in the response to cholinergic pathway activation in this autoimmune model.

The vagus nerve innervates the pancreas and controls both endocrine and exocrine secretions (43, 44). Secretion of ACh *via* vagus nerve stimulation acts on mAChRs expressed on β cells and upregulates the production of insulin (45–48). Our results showing that inhibition of AChE induced an increase in pancreatic insulin mRNA expression are consistent with these previous findings. The induced increase in insulin synthesis could compensate for the damage triggered by STZ and prevent the initial development of hyperglycemia. Moreover, it has been shown that STZ treatment upregulated the expression of pancreatic AChE, which in turn induced the death of islet cells (49). Therefore, the prophylactic inhibition of AChE could prevent STZ-induced destruction of β cells and preserve the production of insulin.

A recent study reported that inhibition of AChE by galantamine delayed the onset of diabetes in NOD mice (50). Moreover, we have preliminary evidence that other AChE inhibitors, such as galantamine and rivastigmine, also prevent the development of hyperglycemia in the MLD-STZ model (data not shown). Increased cholinergic activity through vagus nerve stimulation also plays an important role in suppressing pancreatic inflammation (51). Previously, we and others reported that peripheral administration of an AChE inhibitor resulted in protective anti-inflammatory manifestations at both the mucosal as well as systemic levels (11, 52). Herein, our results show that inhibition of AChE in STZ-treated mice leads to reduced islet infiltration by CD3^+^ T cells and significantly lower levels of pancreatic IL-1β and splenic IL-6 and IL-17 mRNA expression. Therefore, our data suggest that cholinergic pathway activation influences the development of hyperglycemia in the MLD-STZ model by acting at both local (pancreas) and systemic immune system levels.

IFNγ was initially considered the cytokine responsible for the pathogenesis of autoimmune diabetes (53, 54). This view changed with the demonstration that IFNγ-deficient NOD mice were still susceptible to insulitis and diabetes, albeit with delayed onset (55). Moreover, splenocytes from diabetic IFNγ^−/−^ NOD mice could fully transfer disease to naïve mice, suggesting the involvement of other T cell subpopulations. Th-17 cells have been described as potent inducers of autoimmune tissue inflammation. An expansion of pathogenic Th17 cells and excess IL-17 production have been implicated in murine and human T1D (24, 25, 31, 32) and other autoimmune diseases (56, 57). In STZ-induced T1D, mice deficient in IL-17R (28) or IL-17 (29) are resistant to STZ-induced diabetes progression and exhibit reduced insulitis and hyperglycemia, indicating that IL-17 plays a pathogenic role in this model. In the NOD mouse model, inhibition of Th17/IL-17 suppressed the development of diabetes (24, 25). The interplay between Th17 and Th1 at the effector phase appears to be a critical determinant of disease onset and progression. In the present study, we demonstrate that inhibition of AChE upregulates IFNγ synthesis in the pancreas and spleen and increases the frequency of IFNγ-positive Th1 cells. Taken together with the findings showing reduced expression of IL-6 and IL-1β, two cytokines involved in the differentiation of Th17 from memory cells (58), cholinergic pathway-induced immunomodulatory effects led to a reduction in the ratio of IL-17-producing Th17 cells and IL-17 cytokine levels in treated mice. These findings suggest an important role for the cholinergic pathway in regulating the development of autoimmune diabetes.

The spleen is a major secondary lymphoid organ where antigen presentation, T cell activation and clonal expansion can take place. It is known that the vagus nerve does not innervate the spleen directly. Instead, the vagus nerve terminates in the celiac ganglion from which the adrenergic splenic nerve projects, innervating the spleen through the release of noradrenaline. Within the spleen, noradrenaline acts on a subset of memory T cells and stimulate the release of ACh from these cells (59). Our data demonstrate that inhibition of AChE reduced the expression of splenic IL-6 and IL-23, two cytokines with critical roles in Th17 development and pathogenicity (60), and upregulated expression of IL-12 and IFNγ. IL-12 is an important immunoregulatory cytokine, being a potent inducer of IFNγ while inhibiting the production of IL-6 and IL-23 (35, 61). Increasing evidence suggests that IFNγ has modulatory effects on immune cells, including IL-17-producing Th17 cells. The development of Th17 cells from naïve cells is potently inhibited by IFNγ and IL-4 (27, 62). The presence of higher levels of IFNγ in the spleen would interfere with the differentiation of naïve T cells into Th17 cells (63), reducing the availability of pathogenic Th17 cells to migrate to the pancreas. Our findings are consistent with earlier studies suggesting an inhibitory role for IFNγ in the development of Th17 cells and T1D in NOD mice (24, 27). At the level of target tissue (pancreas), inhibition of AChE led to a reduction in IL-1β and IFNγ expression, two proinflammatory cytokines known to induce apoptosis in the pancreatic β cells (64). These findings support the idea that IFNγ in diabetes may have dual functionalities, a pathogenic role at the level of pancreas and a protective role at the level of spleen (24).

A few studies have addressed the direct effect of cholinergic stimulation on isolated immune cell populations. Nicotine, a cholinergic agonist of nAChR, was shown to increase the production of IL-12 by macrophages and DCs *in vitro* (65, 66). Both mAChR and nAChR are known to be expressed on T cells, with muscarinic stimulation leading to enhanced IL-10 and IL-17 production while nicotinergic stimulation upregulating IFNγ and inhibiting IL-17 (67). Therefore, cholinergic stimulation could act directly on T cells to stimulate IFNγ production or indirectly through DCs and macrophages increasing the production of IL-12. The fact that we observed a partial reduction in IL-17-expressing CD4^+^ T cells in paraoxon/STZ-treated IFNγ^−/−^ mice suggests that IL-12 could regulate Th17 activation independent of IFNγ. In addition to Th17 cells, IL-17-producing CD8^+^ T cells (Tc17) are also induced in the MLD-STZ diabetes model (28). Interestingly, the increased frequency of Tc17 cells was only evident early after STZ administration (day 6) but normalized by day 11 post-STZ (28). In our current study, we could not detect any significant presence of Tc17 cells in any of the experimental groups, most likely due the earliest time point being at day 10 post STZ. Nevertheless, a small but significant decrease in total CD8^+^ T cells was noted at day 10 in paraoxon/STZ-treated mice in comparison to the saline/STZ group (Image 2 in Supplementary Material). Therefore, it is possible that administration of AChE inhibitors could also influence disease development by preventing the activation and differentiation of CD8^+^ T cells and Tc17 cells. Furthermore, there is evidence suggesting that IL-12 could enhance the suppressive activity of Tregs, thereby contributing indirectly to the control of pathogenic autoimmune T cells (68). Further studies are needed to investigate the exact mechanism by which AChE inhibition mitigates against the development of pathogenic Th17 cells in autoimmune diabetes.

In conclusion, the findings in the present report demonstrate that inhibition of AChE reduces the incidence of STZ-induced T1D and provides, for the first time, mechanistic evidence that this is achieved through the inhibition of pathogenic Th17 cells. Furthermore, the data demonstrate a pivotal role for IFNγ in mediating the observed protective effect. The possibility of modulating disease severity by therapeutic administration of AChE inhibitors is currently under investigation. Taken together, these results suggest a promising strategy for preventing T1D and other autoimmune diseases.

## Prior Presentation

Parts of the study were presented in abstract form at the 102nd Annual Meeting of the American Association of Immunologists, New Orleans, LA, USA. May, 2015.

## Author Contributions

JG performed all animal experiments and analyzed data. GB performed all histological experiments and analyzed data. MQ helped with obtaining some of the data. YM provided valuable support for all molecular studies. JA contributed to the discussion and reviewed the final manuscript. Ba-R designed the study, analyzed data, and wrote the final manuscript. MF-C designed the study, supervised the project, and wrote the final manuscript.

## Conflict of Interest Statement

The authors declare that the research was conducted in the absence of any commercial or financial relationships that could be construed as a potential conflict of interest. The reviewer IC declared a shared affiliation, though no other collaboration, with one of the authors JA to the handling Editor, who ensured that the process nevertheless met the standards of a fair and objective review.
