# Supplementary material for: Cholinergic Stimulation Prevents the Development of Autoimmune Diabetes: Evidence for the Modulation of Th17 Effector Cells via an IFNγ-Dependent Mechanism
Source: Front Immunol. 2016 Oct 13;7:419. doi: 10.3389/fimmu.2016.00419 (PMC5061850; doi:10.3389/fimmu.2016.00419)
Supplement: Supplementary file 1 [file Data_Sheet_1.PDF]

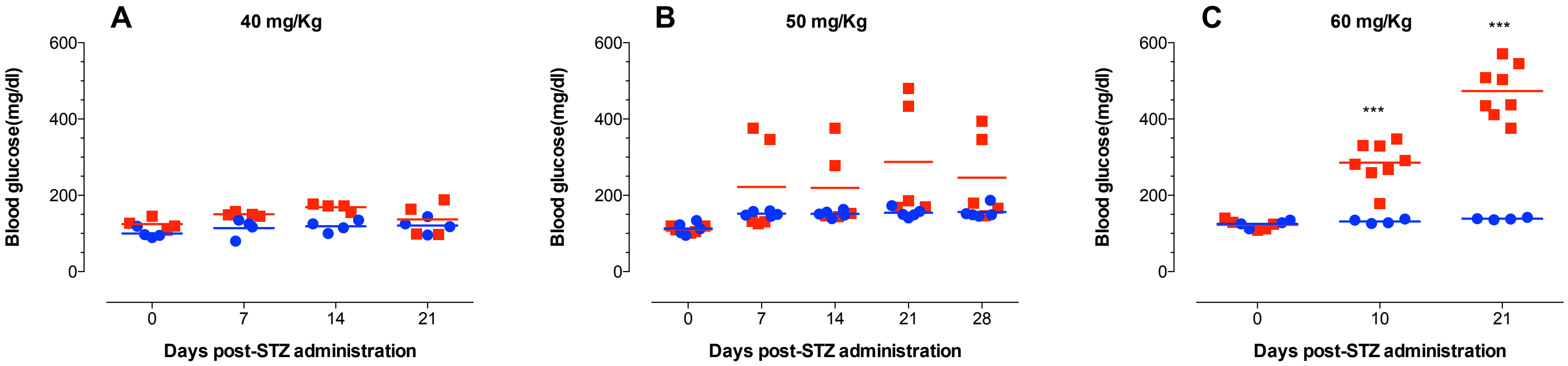

sFig. 1. Optimal dose of STZ to induce experimental hyperglycemia in C57BL/6 mice. C57BL/6 mice were injected with vehicle (blue symbols) or STZ (red symbols) at (A) 40mg/kg, (B) 50mg/kg or (C) 60mg/kg doses. Tail blood samples were analyzed for glucose concentration at the indicated days after STZ treatment. Blood glucose measurements ≥200 mg/dl were considered diabetic. Two-way ANOVA was used for statistical analysis for all graphs. Asterisks denote statistically significant differences between blood glucose levels of STZ and control groups at each time point (****p* ≤0.001).

Supplementary Figure 1

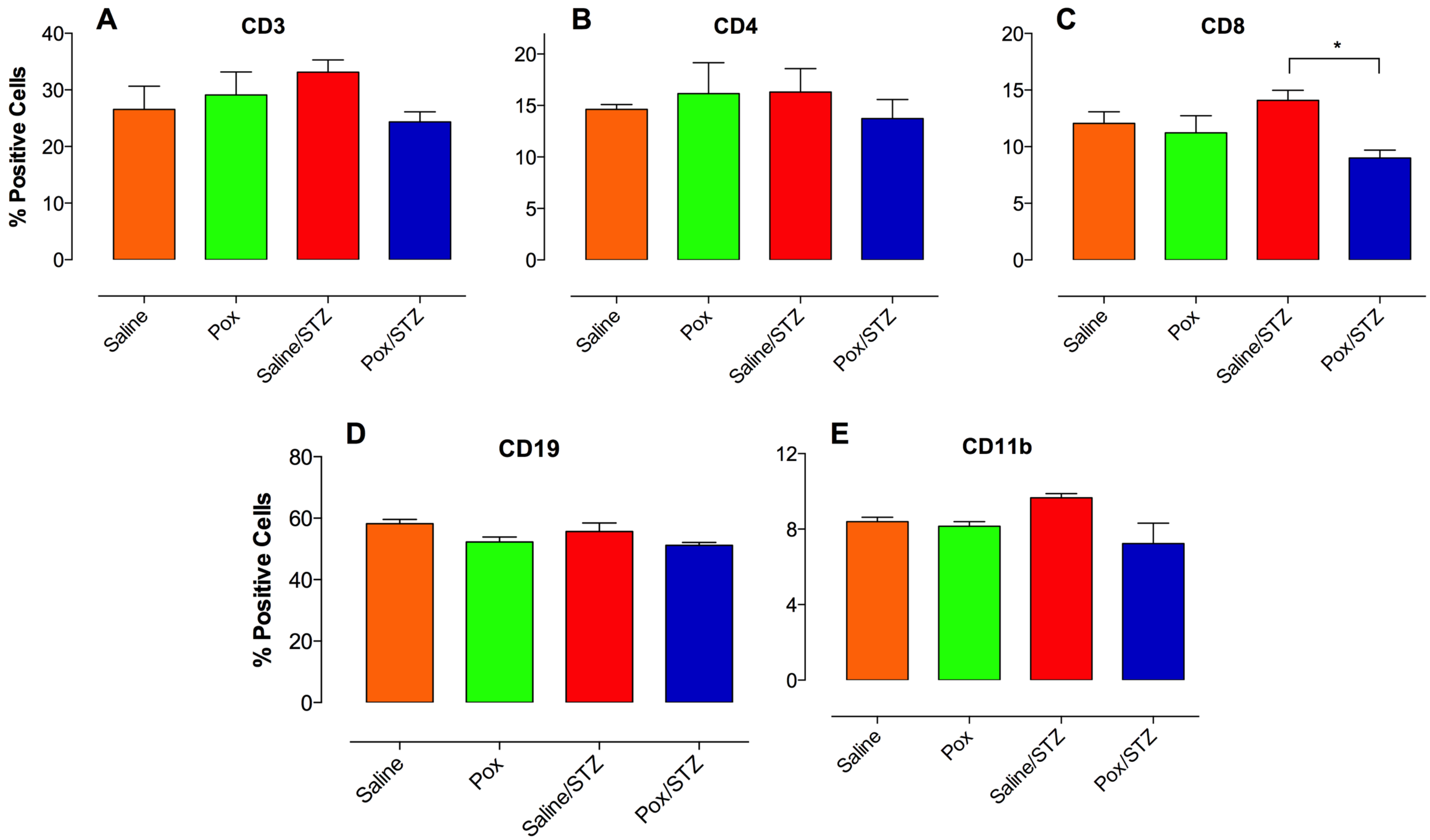

sFig. 2. Immunophenotyping analysis of spleen cell populations at day 10 post STZ administration. Cells were harvested from each of the experimental groups (3 mice/group) and analyzed using a 6-color panel of mAbs specific to CD3, CD4, CD8, CD11b, and CD19. Non-viable cells were gated out by virtue of staining with 7AAD. The bar graphs (panels ***A-E***) depict the percent positive cells (mean ± SEM) in each spleen sub-population. The data is representative of 2 independent experiments. One-way ANOVA was used for statistical analysis for all graphs. Asterisks denote significant differences between the indicated experimental groups (*p <0.05).

Supplementary Figure 2

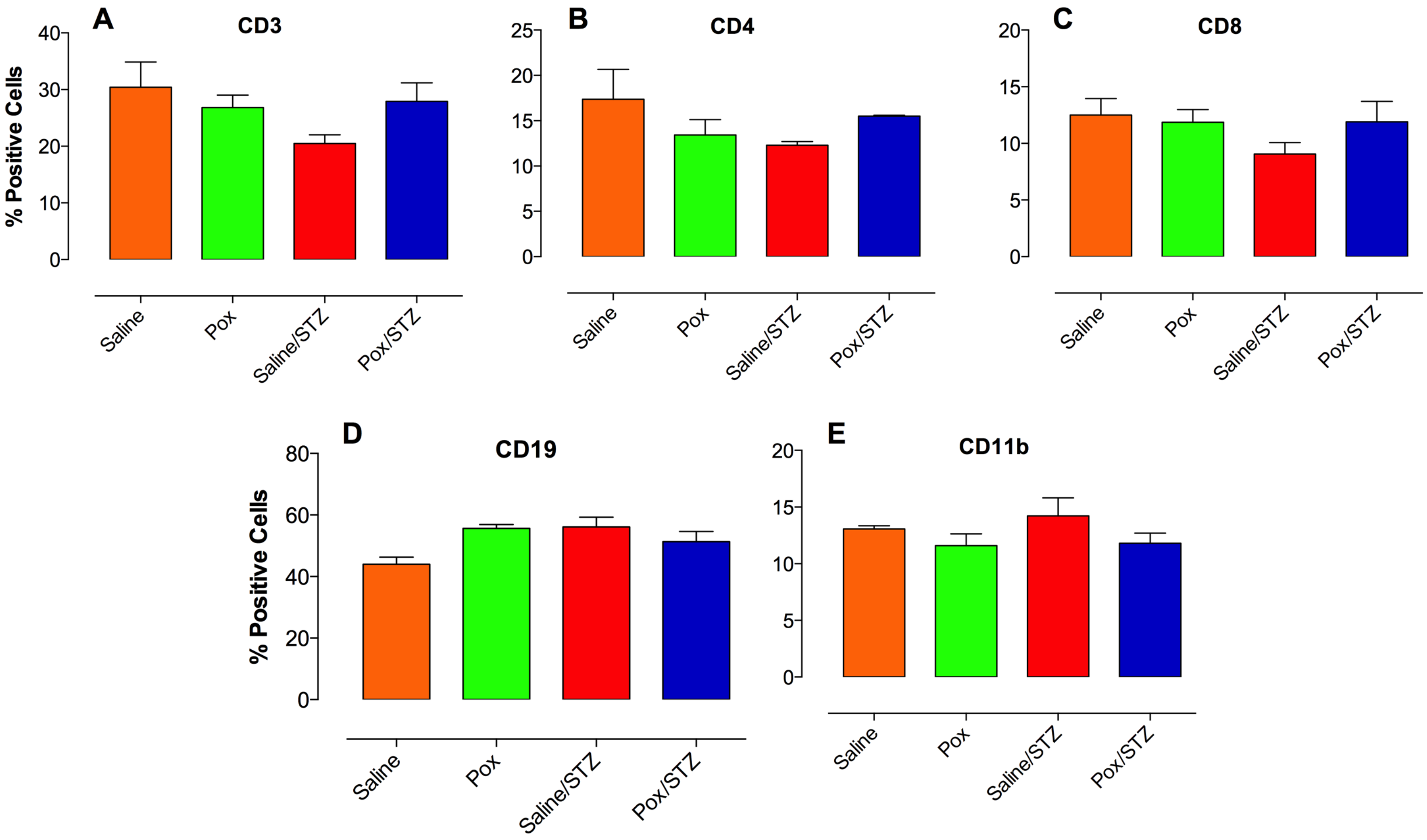

sFig. 3. Immunophenotyping analysis of spleen cell populations at day 18 post STZ administration. Cells were harvested from each of the experimental groups (3 mice/group) and analyzed using a 6-color panel of mAbs specific to CD3, CD4, CD8, CD11b, and CD19. Non-viable cells were gated out by virtue of staining with 7AAD. The bar graphs (panels ***A-E***) depict the percent positive cells (mean ± SEM) in each spleen sub-population. The data is representative of 2 independent experiments. One-way ANOVA was used for statistical analysis for all graphs.

Supplementary Figure 3
